# *Ab initio* reconstruction from one-dimensional crystal diffraction data

**DOI:** 10.1107/S2053273322001942

**Published:** 2022-04-05

**Authors:** Romain D. Arnal, Rick P. Millane

**Affiliations:** a Computational Imaging Group, Department of Electrical and Computer Engineering, University of Canterbury, Christchurch, New Zealand

**Keywords:** 1D crystals, phase problem, iterative projection algorithms, shrink-wrap algorithm, filaments

## Abstract

Filamentary and rod-like assemblies are ubiquitous in biological systems, and single such assemblies can form one-dimensional (1D) crystals. New, intense X-ray sources, such as X-ray free-electron lasers, make it feasible to measure diffraction data from single 1D crystals. Such experiments would present some advantages, since cylindrical averaging of the diffraction data in conventional fiber diffraction analysis is avoided, there is coherent signal amplification relative to single-particle imaging, and the diffraction data are oversampled compared with those from a 3D crystal so that the phase problem is better determined than for a 3D crystal [Millane (2017). *Acta Cryst.* A**73**, 140–150]. Phasing of 1D crystal diffraction data is examined, by simulation, using an iterative projection algorithm. *Ab initio* phasing is feasible with realistic noise levels and little envelope information is required if a shrink-wrap algorithm is also incorporated. Some practical aspects of the proposed experiments are explored.

## Abstract 2

Methods for reconstructing electron densities from diffraction amplitudes alone, measured from single 1D crystals, are described and illustrated by simulation. *Ab initio* phasing is demonstrated using an iterative projection algorithm.

## 1. Introduction

Molecules forming filaments, fibers or, in general, high-aspect-ratio rod-like assemblies, have been studied as a class of their own, alongside globular and membrane proteins, since the beginning of the field of structural biology. Indeed, X-ray diffraction patterns from DNA fibers, dating back to 1952, offered a first look at the genetic information storage of DNA, and are a prime example of these commonly occurring targets and their significance (Gosling, 1954 ▸). Filamentary structures are the natural way that biomolecules can assemble with equivalent contacts along an axis, to build many of the structures of cells (*e.g.* microfilaments, microtubules, myofilaments) (Erlandson, 1989 ▸; Stubbs, 1999 ▸). Furthermore, equivalent contacts between a variety of subunit structures can be achieved by the filaments adopting helical symmetry (Diaz *et al.*, 2010 ▸). Studies of the structures of filamentary assemblies are therefore of key importance in structural biology.

The first main approach to structure determination for filamentary assemblies is so-called X-ray fiber diffraction analysis (Stubbs, 1999 ▸; Millane, 2010 ▸). In this approach, an oriented fiber specimen of the molecule of interest is prepared, its X-ray diffraction pattern is measured, and the diffraction data used as the basis for structure determination. The hallmark of a fiber specimen is that it contains a large number of molecules that are oriented with their long axes approximately parallel. Alignment must be within a few degrees for high-resolution studies. The oriented molecules may assemble, side by side, in a random manner, in a so-called noncrystalline fiber, or they may assemble into small crystallites which, in turn, assemble side by side in a random manner, in a so-called polycrystalline fiber. The second hallmark of a fiber specimen is that either the individual molecules (in a noncrystalline fiber) or the crystallites (in a polycrystalline fiber) are randomly rotated about the orientation axis. This rotational disorder in the specimen results in the measured diffraction being cylindrically averaged about the corresponding axis in reciprocal space. The cylindrical averaging substantially reduces the information content of the diffraction data relative to that from a single crystal or from a single particle. The reduced information content makes structure determination difficult since one has to, in effect, unravel the cylindrical averaging in addition to solving the usual phase problem (Stubbs, 1975 ▸; Namba & Stubbs, 1985 ▸; Millane, 2010 ▸).

Despite the challenges of X-ray fiber diffraction analysis, it has played an important role in determining the structures of many such assemblies (Stubbs, 1999 ▸; Millane, 2010 ▸). It is, however, technically demanding, depending on model building for molecules with small repeating units, or collection of additional diffraction data for larger assemblies, or is limited to low resolution. As a result, the application of fiber diffraction analysis has been quite limited relative to single-crystal crystallography, and there are many fibrous systems in biology that await structural analysis.

The second important technique for studying the structures of filamentary assemblies is cryo-electron microscopy (cryo-EM) (De Rosier & Klug, 1968 ▸; Egelman, 2000 ▸; Yonekura *et al.*, 2003 ▸; Unwin, 2005 ▸). With the advent of direct electron detectors and advances in image processing methods, cryo-EM has become the method of choice for high-resolution structural studies of fibrous assemblies (Fromm *et al.*, 2015 ▸; Gutsche *et al.*, 2015 ▸; DiMaio *et al.*, 2015 ▸; Fromm & Sachse, 2016 ▸; Fitzpatrick *et al.*, 2017 ▸; Bradshaw & Paul, 2019 ▸). Despite these improvements in cryo-EM, X-ray imaging of these targets has key advantages when room-temperature dynamics or time-resolved aspects of the structure are important, or in cases where cryo-EM fails.

Zuo *et al.* (2003 ▸) reported imaging of a double-wall carbon nanotube using electron diffraction data and performing phase retrieval. They used a single 2D diffraction pattern and reconstructed a projection image, and the specimen was not strictly periodic so the procedure was more analogous to single-particle imaging.

The fundamental difficulty of X-ray fiber diffraction analysis is one of signal level. Many molecules in the specimen are required to produce measurable diffraction, but the rotational disorder results in cylindrical averaging of the diffraction. These difficulties would be overcome if diffraction could be measured from a single molecule, since there is then no cylindrical averaging. High-intensity X-ray free-electron laser (XFEL) sources with sub-micron focal spots offer the opportunity to measure diffraction from single fibrillar assemblies, while also avoiding radiation damage, thus circumventing the primary difficulties of cylindrical averaging and disorientation in fiber diffraction analysis, for determining the structures of these molecules. Fibrous assemblies are frequently periodic along their long axis, *i.e.* are one-dimensional (1D) crystals, which is the case considered here. If data from such specimens are measured, then the phase problem for 1D crystals takes on practical importance. In particular, diffraction data from a 1D crystal are rich compared with those from a 3D crystal, and there are significant opportunities for *ab initio* phasing (Millane, 2017 ▸). 1D crystals present an advantage over single-particle (noncrystalline) diffraction due to Bragg amplification boosting the signal level.

Diffraction experiments with filaments, fibrils and fibers using XFELs have recently been reported. Popp *et al.* (2017 ▸) measured diffraction patterns at 10–20 Å resolution from four filament specimens flow-aligned in a liquid micro-jet, with at least 100 individual filaments in the beam. However, the random rotation of the filaments in the beam focus meant that the data were still cylindrically averaged as in conventional diffraction patterns from fibers. Branden *et al.* (2019 ▸) used data of this kind from flow-aligned collections of about 20 microtubules to obtain 2D projections of the electron density at 20 Å resolution, *i.e.* the 2D projection is recoverable from the cylindrically averaged diffraction data. Seuring *et al.* (2018 ▸) recorded diffraction to 2.4 Å resolution from amyloid protofibrils, and from tobacco mosaic virus (TMV) filaments to 2.7 Å, mounted and aligned on graphene, with, in some cases, only a few amyloid protofibrils or TMV filaments in the XFEL focus. This method of sample support on ultra-clean graphene offers a dramatic reduction in background diffraction compared with liquid-jet delivery. On average, about eight TMV filaments and 50 amyloid protofibrils were sampled in the 150 nm focal diameter of the XFEL beam. Some patterns with only a few fibrils in the beam were also recorded. Finally, Wojtas *et al.* (2017 ▸) recorded weak diffraction patterns from individual crystalline amyloid fibrils delivered and oriented in a liquid jet, that were analyzed, re-oriented and merged into a 3D data set. This experiment demonstrates the feasibility of measuring diffraction from single, tiny crystalline fibrils, and increasing the signal-to-noise ratio (SNR) by orientating and averaging the weak patterns. In this case, however, the fibrils are essentially single 3D crystals, giving Bragg diffraction, and the resulting phase problem is identical to that in conventional single-crystal crystallography, and so does not accrue the advantages of the increased information content of diffraction by single 1D crystals that is considered here.

In light of these results, it is likely that future developments in source brightness and pulse rates, sample preparation and delivery methods, detectors and data processing algorithms, will allow diffraction data from single filaments to be collected, oriented, merged and used for structure determination. In the absence of cylindrical averaging, and with the increased diffraction sampling from single 1D crystals, *ab initio* phasing is possible. Methods for *ab initio* electron-density reconstruction from such data are explored in this paper.

Latychevskaia & Fink (2018 ▸) have investigated direct imaging of helical molecules from both 3D, and cylindrically averaged 2D, diffraction patterns. For 3D data, analogous to the case considered here, they demonstrated reconstruction of point double helices from simulated data, although general electron-density reconstruction was not considered. They also demonstrated low-resolution reconstruction from cylindrically averaged data, although that is beyond the scope of the current paper.

Key results concerning the phase problem for 1D crystals (Millane, 2017 ▸) are reviewed in Section 2, and in Section 3 the iterative projection algorithms that we use for phase retrieval are briefly reviewed. In Section 4, our phase retrieval algorithm for *ab initio* reconstruction from 1D crystal diffraction data is described. Simulation results are presented in Section 5. Some practical considerations in implementing these ideas in XFEL imaging are discussed in Section 6, and concluding remarks are made in Section 7.

## 2. 1D crystal phase problem

Uniqueness properties of phase problems in various forms have been studied extensively (Elser & Millane, 2008 ▸; Millane, 2017 ▸; Arnal & Millane, 2017 ▸). Uniqueness of the solution to a phase problem is conveniently described with the help of the constraint ratio, denoted Ω, which is equal to the ratio of the number of independent intensity data available to the number of independent parameters describing the object. If, for a particular problem, 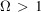 , then the problem is highly constrained and a unique solution (or a small number of solutions) is expected. In practice, as a result of noise and missing data, and other uncertainties, an additional margin will be needed. If 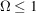 , the solution to the phase problem is not unique and a multitude of objects are consistent with the intensity data and real-space constraints.

It is easily shown that for a single object 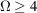 (Elser & Millane, 2008 ▸), and therefore a unique solution to the phase problem is expected in general. The phase problem then, at least, is not a significant impediment for single-particle imaging. For a 3D crystal however, as a result of the reduced data set that is restricted to the intensities of the Bragg reflections, 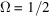 , or 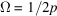 if the protein content *p* of the crystal is known (Millane & Arnal, 2015 ▸). Therefore, except in the case of high-solvent-content crystals, and in the absence of additional real-space constraints, 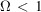 , and the crystallographic phase problem is highly non-unique. Additional data or information are therefore needed to provide some phase information. Additional constraints, such as non-crystallographic symmetry, may render the solution unique. For a 2D crystal, 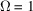 in general, but 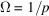 if some envelope information is available (Arnal & Millane, 2017 ▸). The 2D crystal phase problem is therefore non-unique in general, although there is some potential for *ab initio* phasing in favorable cases.

Let us return now to the case at hand of a 1D crystal. Uniqueness properties of the phase problem for a 1D crystal are described in detail by Millane (2017 ▸) and are summarized here. Diffraction from a 1D crystal consists of planes, so-called layer planes, in reciprocal space. (Note that in the case of fiber diffraction, the cylindrical averaging reduces the layer planes to so-called layer lines.) The planes are spaced by the Bragg spacing, and the diffraction intensity can be measured, in principle, continuously on each layer plane. As a result of the increased sampling relative to the full 3D Bragg sampling from a 3D crystal, 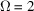 in general for a 1D crystal. However, although this favorable constraint ratio indicates that the problem is highly constrained, as a result of the highly structured sampling by the layer planes, many solutions are still permitted (Millane, 2017 ▸). The solution set is low dimensional relative to the dimensionality of all electron densities, but still forms a large set of possible solutions. However, the low dimensionality means that minimal additional *a priori* information is needed to render the solution unique. In particular, if the molecular envelope is known and deviates from a cylinder (of any cross section) then the solution is highly constrained (Millane, 2017 ▸). Millane (2017 ▸) shows that the quantity 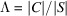 , where 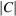 denotes the volume of the smallest cylinder that circumscribes the molecule and 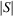 denotes the volume of the molecular envelope, describes the constraining power of the envelope, in the sense that 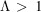 ensures a unique solution. Larger deviations of the envelope from a cylinder, and then larger values of Λ, correspond to a more constrained solution.

In summary then, the phase problem for a 1D crystal admits multiple solutions, but a unique solution is expected in the presence of minimal envelope information. Interestingly, known helix symmetry, often present in 1D crystals, does not provide additional constraints on the 1D crystal phase problem (Millane, 2017 ▸).

## 3. Iterative projection algorithms

Iterative projection algorithms (IPAs) have been used successfully to solve phase problems by reformulating them as a constraint satisfaction problem. We briefly describe here the IPA that we use for reconstruction from 1D crystal diffraction data, and for the simulations described in the next section. See Millane & Lo (2013 ▸) for more information on IPAs.

IPAs take the approach of representing the problem as a constraint satisfaction problem in which a solution is sought that satisfies two constraints. In crystallographic phase retrieval, the two constraints are in reciprocal space and real space. The reciprocal-space constraint imposes the condition that the amplitude of the Fourier transform of the electron density is equal to that measured. The real-space constraint imposes known properties of the electron density, such as a known molecular envelope, positivity *etc*. We denote by 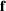 the vectorized electron density in the unit cell, which belongs to the vector space 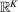 , and represents all possible densities with *K* samples in the unit cell. We denote by 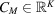 the set of all densities that satisfy the reciprocal-space constraints, and by 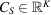 the set of all densities that satisfy the real-space constraints. The solution to the phase problem is then any point in the intersection 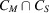 .

IPAs search the vector space 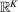 for a point in the intersection, and use projection operators. A projection makes the smallest change to a point in the vector space (an electron density) to give a new point (density) that satisfies the constraint. Mathematically, the projection 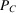 of the vector 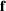 into a constraint set *C*, denoted 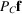 , is given by 

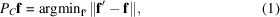

where 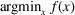 denotes the value of *x* that minimizes 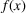 and 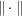 denotes the Euclidean norm. So-called relaxed projections are also used in IPAs; the relaxed projection, denoted 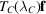 , is given by 

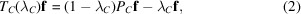

where 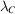 is called the relaxation parameter. The use of relaxed projections in IPAs can improve convergence.

An IPA generates a sequence of iterates 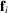 , starting from a random electron density 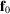 , that ideally converges to a solution (a point in the intersection). A particular IPA is defined by an update rule, which is the operation, made up of projections, that takes 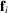 to 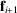 . Various IPAs are in use, and here we use the difference map (DM) algorithm (Elser, 2003 ▸) that has good convergence properties for non-convex constraints (as is the constraint 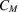 ). The update rule for the DM algorithm is given by 

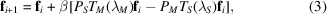

where 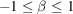 is a parameter and 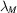 and 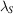 are usually set to 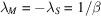 . The parameter β is then the only parameter of the DM algorithm. The value of β used can affect the speed of convergence, but the choice of value is generally not particularly critical. A value between 0.5 and 1 is often used, although sometimes negative values are also used.

Note that the iterate is not itself an estimate of the solution, and that once the algorithm has converged, or reached a fixed point, *i.e.* 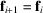 , the solution 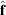 (that satisfies both constraints) is given by (Elser, 2003 ▸; Millane & Lo, 2013 ▸) 

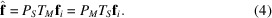




## 4. Phase retrieval

Reconstruction of the electron density from simulated 1D crystal data is used to investigate the feasibility of *ab initio* phase retrieval with minimal *a priori* information, using the DM algorithm. Realistic noise levels for XFEL data from single 1D crystals are considered. The phase retrieval algorithm and simulation methods are described here.

The Fourier amplitude data are calculated as for a 1D crystal, *i.e.* Bragg sampled in the axial dimension, and oversampled by a factor 3 in the two lateral dimensions. Various initial envelopes are considered. The DM algorithm is used with 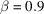 , and the algorithm is started with a random electron density inside the starting envelope. A positivity constraint is applied along with the envelope (support) constraint. The Fourier amplitude constraint is applied for all measured, *i.e.* observed, intensities, unmeasured low-resolution intensities are allowed to float, and high-resolution intensities beyond the resolution limit are set to zero.

In many of the simulations, the shrink-wrap algorithm (Marchesini *et al.*, 2003 ▸) is used in conjunction with the DM algorithm. The shrink-wrap algorithm is used to improve the envelope as the iterations proceed, and it plays a key role in evolving the envelope away from a cylindrical starting envelope.

We consider a 1D crystal with period (cell constant) *c*. The value of *c* is easily determined from the diffraction data. As described in Section 1, molecules that form 1D crystals frequently possess helical symmetry. The molecular symmetry is a discrete symmetry, with *u* subunits in *v* turns of the helix in one *c* repeat, being referred to as 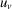 -helix symmetry. The values of *u*, and usually of *v*, are straightforwardly determined from the diffraction data (Millane, 2010 ▸). Any particular element of the structure (atom, residue *etc*.) then lies on a filamentary helix of pitch 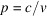 . The helix pitch can then be considered known, up to any possible ambiguity in the value of *v*.

Frequently, although not always, as a result of the molecular helix symmetry, there is a region on the periphery of the molecule, forming a continuous helix, or screw, which is completely outside the molecule and in which the electron density is zero. This is often referred to as a helical groove. B-DNA, for example, has two such grooves, referred to as the major and minor grooves, with the major groove being quite deep. Knowledge of the pitch and the possible existence of a groove can be used as a support constraint and help to constrain the envelope away from a cylinder.

For the purposes of a helical envelope constraint, although the pitch of the helix is known, the hand (left- or right-handed) may be unknown. If the incorrect hand is chosen for the envelope, then a good reconstruction will be obtained that is the enantiomer of the correct structure. For biological molecules, the hand of the residues will be incorrect, and the correct density is obtained by inverting the reconstruction and reversing the hand of the helical envelope.

It is important to note that any discrete molecular helix symmetry does not constrain the phase problem in this case (as noted above), and any such symmetry is not used or imposed in the reconstructions. However, the presence of a continuous helical groove of known pitch in the envelope does constrain the solution, since the envelope is then not cylindrical. Considering a helically symmetric envelope provides a useful simplification, and is used here to simplify application of the shrink-wrap algorithm, as is described below.

Although the shrink-wrap algorithm is generally applied in 3D, here we constrain the envelope to have continuous helical symmetry of known pitch, and are thus able to apply the shrink-wrap algorithm in 2D. This approach slightly restricts the available envelopes, but it considerably simplifies the implementation. The shrink-wrap is achieved by untwisting and then projecting the electron density, giving the projected untwisted density denoted 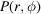 as 

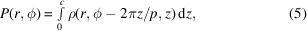

where 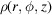 is the electron density in cylindrical polar coordinates 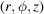 . The 2D shrink-wrap is applied to 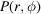 , giving a 2D envelope denoted 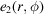 , which is then expanded out to a 3D envelope 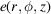 as 

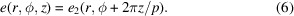

Note that the cylindrical envelope is defined by the pitch *p*, and not by the discrete helical symmetry parameters *u* and *v*. Note also that although a 2D continuous helical envelope is used, the electron density is reconstructed in 3D within the envelope, and the density itself is not constrained to have helical symmetry.

Shrink-wrap is applied in the usual way (Marchesini *et al.*, 2003 ▸) by first blurring the projected density with a 2D Gaussian and then thresholding the resulting density to form a new 2D envelope, which is retwisted to form the new 3D envelope as described above. The width of the Gaussian used is problem dependent and a full width at half-maximum of 10 Å (approximately three times the resolution of the data) was used in the simulations. In the simulations, the initial threshold is set to zero and is incremented by a fixed value of 0.5σ, where σ is the standard deviation of the electron density, at each shrink-wrap step. If increasing the threshold would cause the new envelope volume to be smaller than the protein volume (assumed known from the estimated solvent content of the crystal), then the increment value is halved. Shrink-wrap is applied every five IPA iterations. The shrink-wrap algorithm is implemented slightly differently to that described by Marchesini *et al.* (2003 ▸) in that the threshold is increased rather than the width of the Gaussian decreased, as the iterations proceed. This approach was found more effective in moving the envelope away from a cylinder.

Three error metrics are calculated to assess convergence of the DM algorithm and the reconstruction quality. The first error metric, similar to the *R* factor, 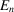 , measures the difference between the measured amplitude data 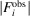 , for datum *i*, and the corresponding Fourier amplitude of the iterate 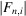 , at iteration *n*, and is given by 

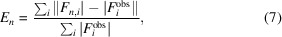

where the sum is over all the measured data *i*.

The second error metric, denoted 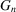 , measures the difference between the true (but unmeasured) low-resolution amplitudes 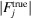 and the corresponding Fourier amplitude of the iterate, *i.e.*


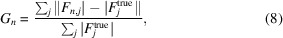

where the sum is over all the unmeasured low-resolution amplitudes *j*. Note that 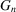 cannot be calculated in practice, but is useful in simulation to see if the low-resolution amplitudes are correctly reconstructed.

The third error metric, 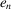 , measures the quality of the reconstruction (electron density), and is given by 

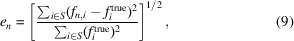

where 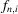 is the electron density at iteration *n* and 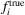 is the true electron density, and the sum is over all the sample points *i* in the envelope *S*.

A successful reconstruction gives small values of all three metrics. Convergence to a small 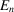 but a large 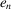 indicates a non-unique solution.

## 5. Simulation results

Three sets of simulations were conducted. The first uses a small synthetic object to illustrate the uniqueness results described in Section 2. The second and third sets use the known molecular structures for DNA, with a relatively small repeating unit, and a recombinase filament, with a larger repeating unit, respectively.

### 5.1. Simple object

**Figure 1:**
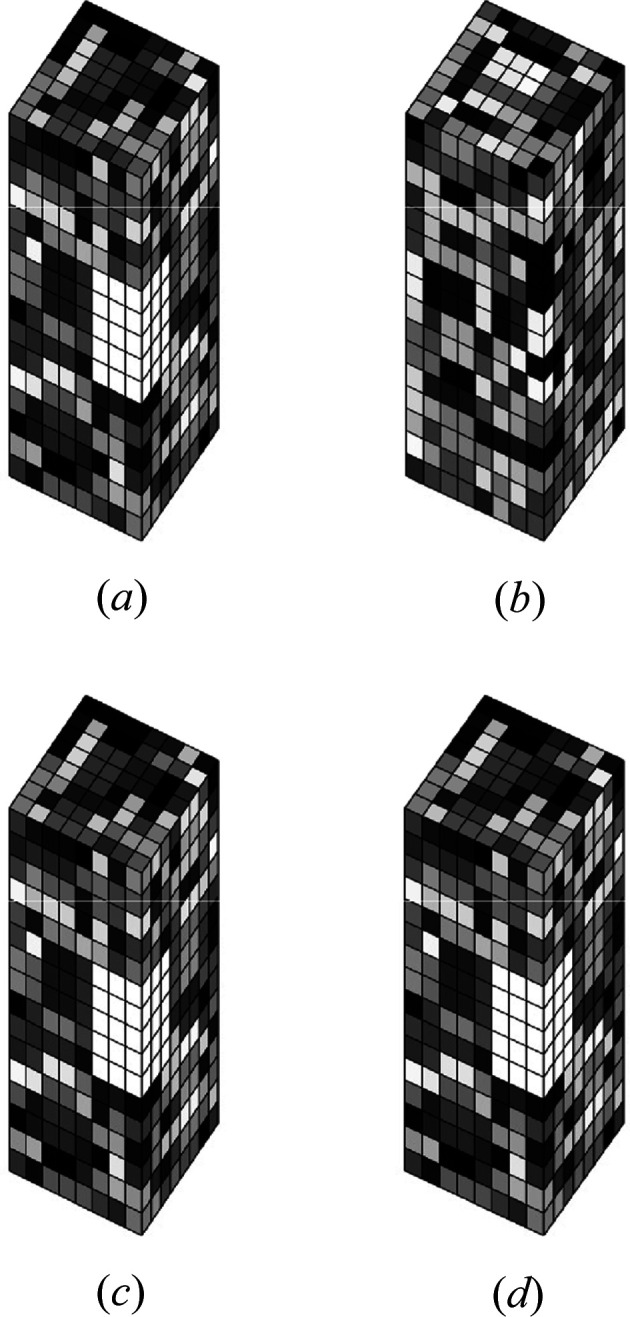
(*a*) True object electron density, and reconstructions for a cylindrical envelope (*b*) without (Case 1) and (*c*) with (Case 2) a positivity constraint, and (*d*) with a non-cylindrical envelope constraint (Case 3), as described in the text.

To illustrate the effect of envelope and positivity constraints on uniqueness of the solution, as described in Section 2, reconstructions of a simple 3D object were conducted. The object has 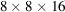 samples, with each assigned a random value from a uniform distribution on (0, 1). To simulate a non-cylindrical envelope, a block of 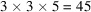 samples on the edge of the object are set to zero (Fig. 1 ▸). The object is zero padded to 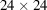 samples in the two lateral dimensions, and 1D crystal, noise-free Fourier amplitude data calculated.

Reconstructions were attempted with the DM algorithm using the Fourier amplitude data for three cases. The first case used the cylindrical ( 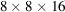 ) envelope, the second used the cylindrical envelope with a positivity constraint, and the third used the non-cylindrical envelope (with the 45 zero-valued samples excluded). The algorithm was run 100 times for each case, with 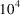 iterations for each run, and the results are summarized in Table 1 ▸. The object and typical reconstructions for the three cases are shown in Fig. 1 ▸. Referring to Table 1 ▸, alternative solutions were obtained for the cylindrical envelope, but either positivity or a non-cylindrical envelope are sufficient to obtain a unique solution. Convergence was quite rare for a positivity-only constraint, but only the true solution was obtained on convergence. For a non-cylindrical envelope, convergence was reasonably frequent. Note that Λ only slightly larger than unity is sufficient for a unique solution in the case of perfect, noise-free data. The results support the uniqueness properties described in Section 2.

### 5.2. B-DNA

**Figure 2:**
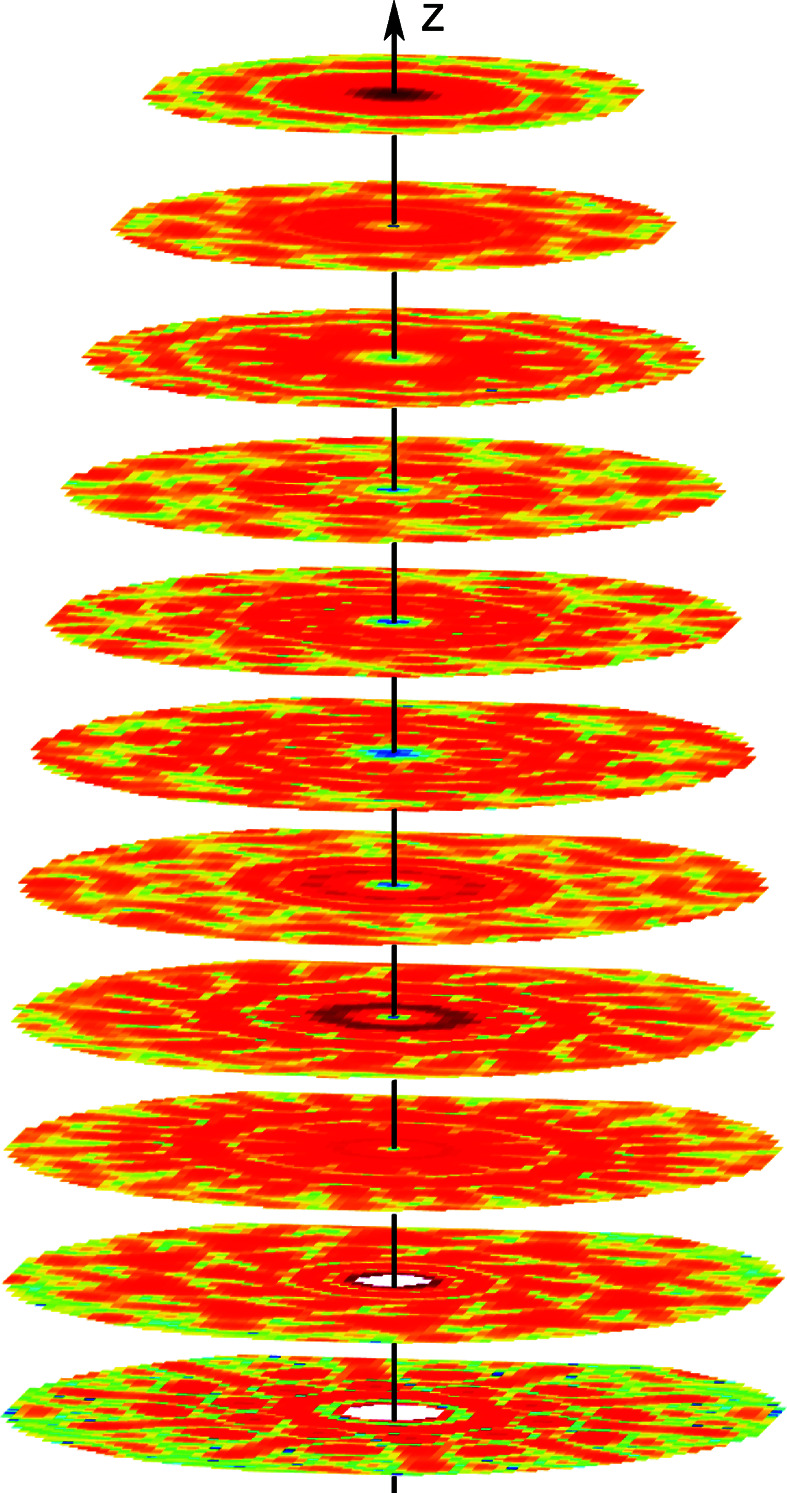
The diffracted intensity (log scale) for a 1D DNA crystal used as data for the simulations. The zeroth layer plane is shown at the bottom and the tenth layer plane at the top.

Structure determination of nucleic acids was one of the first beneficiaries of X-ray fiber diffraction analysis. We use the molecular structure of B-DNA to examine phase retrieval from 1D crystal diffraction data. The structure used is the synthetic polynucleotide poly(dA)·poly(dT) (Chandrasekaran *et al.*, 1995 ▸), PDB entry 1ply, which has a *c* repeat and pitch of 32.4 Å, 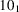 -helix symmetry and an outside cylindrical radius of 10 Å. Note that this is a synthetic DNA with adenine on one strand and thymine on the other strand. Diffraction data were calculated between 20 and 2.5 Å resolution, with Gaussian noise added to the diffraction intensities such that 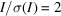 in the highest-resolution shell between 3 and 2.5 Å, and used for the reconstructions. The diffraction data on the layer planes are depicted in Fig. 2 ▸. The DM algorithm was run ten times with 1000 iterations for each run.

**Figure 3:**
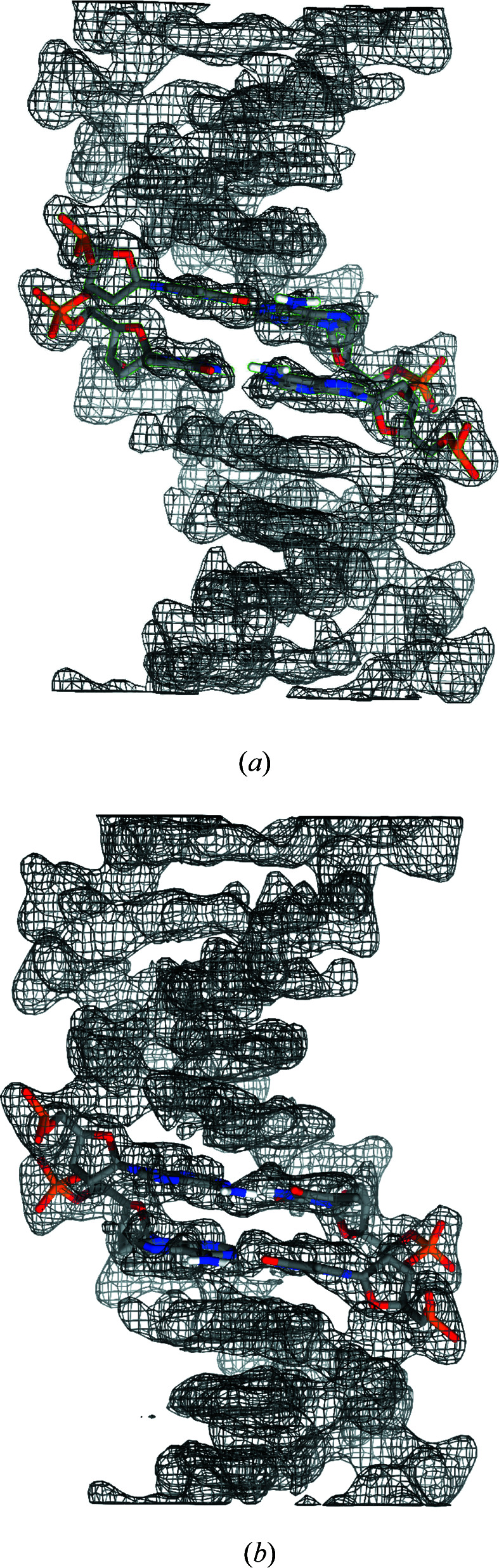
Reconstructed electron density of one *c* repeat of B-DNA (*a*) with a fixed envelope consisting of a circular cylinder with a helical groove of volume 10% of the cylinder, and (*b*) with a starting cylindrical envelope with the 2D shrink-wrap algorithm applied. Two base pairs of DNA are also shown. Figure obtained using *Chimera* (Pettersen *et al.*, 2004 ▸) with electron density cut at 1σ.

The first set of simulations used a fixed envelope. The envelope was either a circular cylinder or a circular cylinder with a helical groove of pitch 32.4 Å, of various sizes, removed. The simulation results are summarized in Table 2 ▸. For a fixed cylindrical envelope the algorithm did not converge. Convergence was also not obtained with a small groove of 5% of the volume of the circumscribing cylinder. For groove volumes greater than 10% of the cylinder volume ( 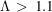 ), good reconstructions were obtained. An example reconstructed electron density for 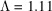 is shown in Fig. 3 ▸(*a*). The two base pairs of the molecular structure shown in the figure indicate a good-quality reconstructed density. These results are consistent with the theory presented in Section 2, particularly given the noise and missing data.

**Figure 4:**
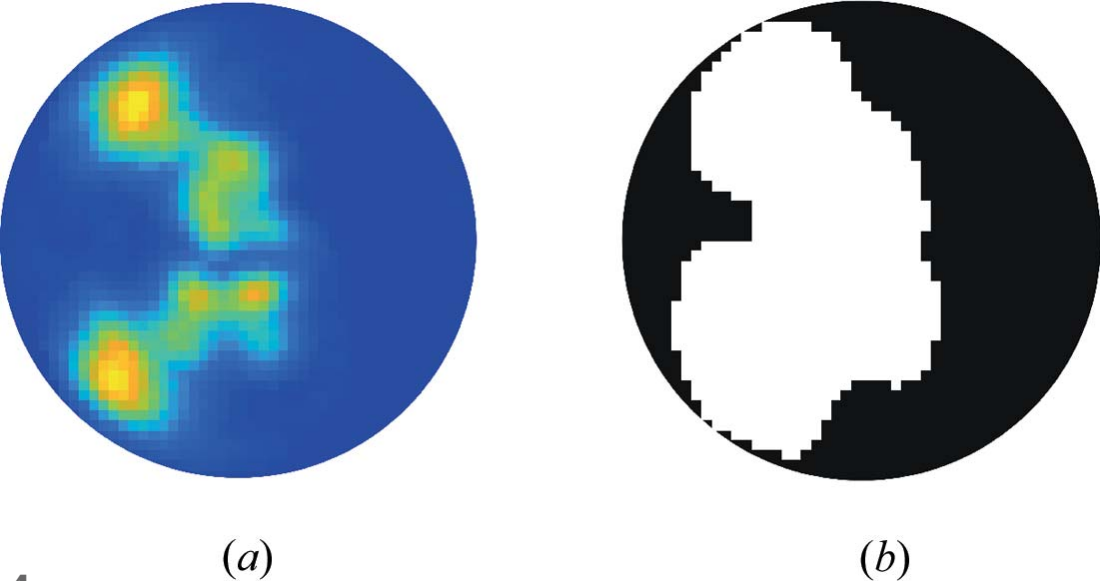
(*a*) The final projected untwisted reconstructed electron density and (*b*) the final 2D envelope, for a B-DNA reconstruction using a starting cylindrical envelope (shown by the circles) and application of the shrink-wrap algorithm.

The second set of simulations used a circular cylindrical starting envelope and application of the 2D shrink-wrap algorithm, as described in Section 4. In this case, in eight out of ten runs, a groove evolved and a good reconstruction was obtained. An example reconstructed electron density is shown in Fig. 3 ▸(*b*). The fractional volume of the final groove was about 55%. The final projected untwisted electron density and the final 2D envelope are shown in Fig. 4 ▸.

The encouraging, and possibly surprising, result is that even with a cylindrical starting envelope, the shrink-wrap algorithm is able to evolve a non-cylindrical envelope that allows a good reconstruction.

### 5.3. RAD51 recombinase filament

The RAD51 recombinase forms a presynaptic filament on single-stranded DNA (Conway *et al.*, 2004 ▸). The inactive structure of this filament has been determined by X-ray crystallography (Conway *et al.*, 2004 ▸), PDB entry 1szp, and is used as the basis for simulation of *ab initio* reconstruction of a large macromolecular assembly from 1D crystal data. The assembly has 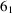 -helix symmetry with a *c* repeat (and pitch) of 130 Å, and an outside radius of 46.5 Å. The assembly has a large groove, although it is proportionally smaller than the major groove of B-DNA. Using the known structure, synthetic diffraction data were calculated between 40 and 3.6 Å resolution and Gaussian noise added to the intensities such that 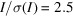 in the highest-resolution shell between 4.1 and 3.6 Å. The DM algorithm was run ten times as described above, starting with a random electron density, and the shrink-wrap algorithm was incorporated into all the reconstructions.

**Figure 5:**
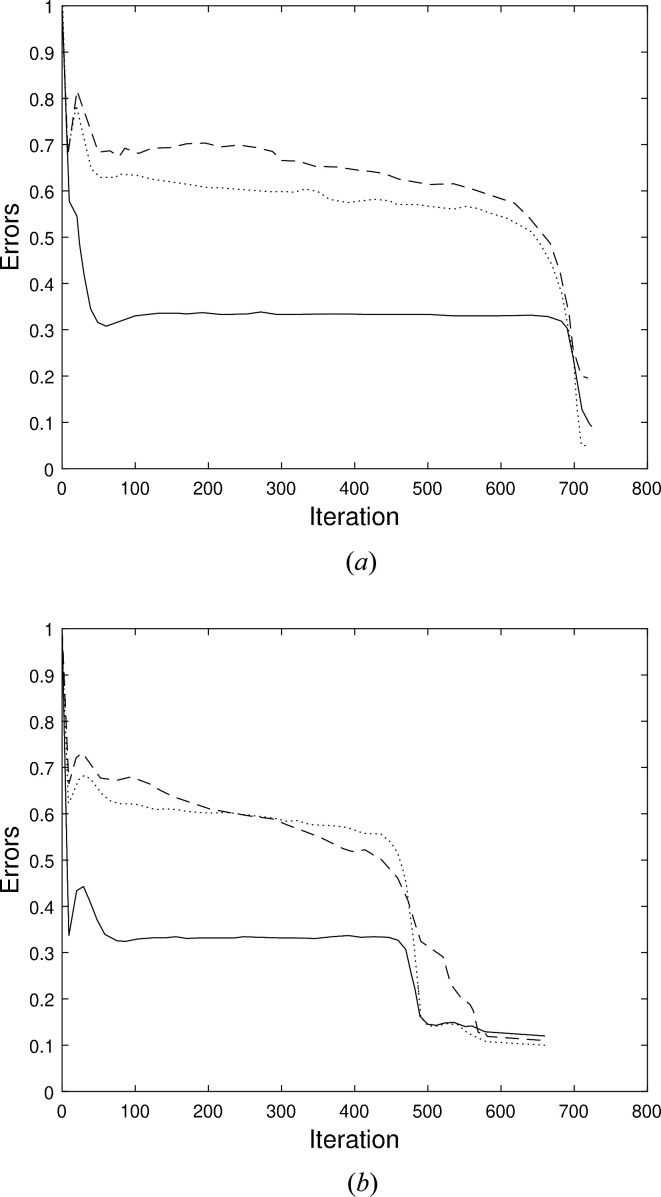
Error metrics *E* (solid), *G* (dots) and *e* (dash) as a function of iteration for an example reconstruction of the electron density of the RAD51 filament, starting with (*a*) a cylindrical envelope, and (*b*) a cylindrical envelope with a groove.

**Figure 6:**
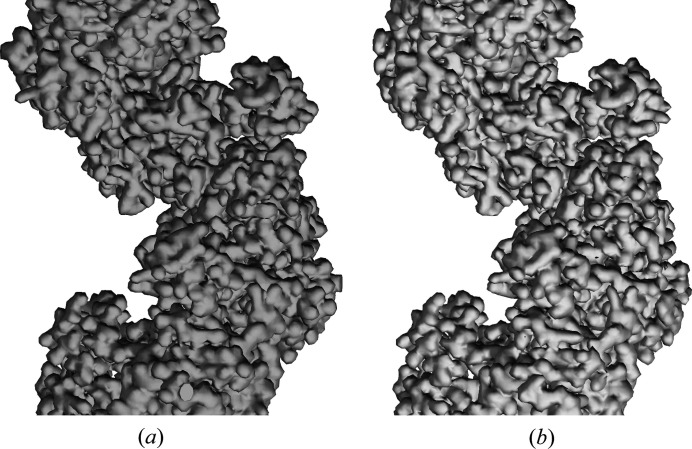
(*a*) True and (*b*) reconstructed (with a starting cylindrical envelope and data in the range 40–3.6 Å) electron density of the RAD51 filament.

**Figure 7:**
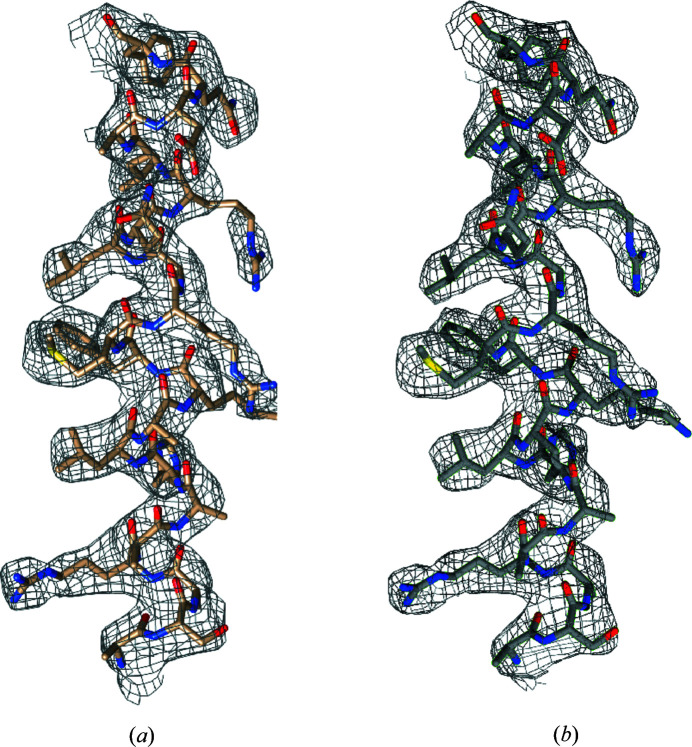
Reconstructed electron density in the region of residues LEU 296 to PHE 317 of the RAD51 filament, (*a*) starting with a cylindrical envelope, and data in the range 40–3.6 Å resolution, and (*b*) starting with a cylindrical envelope with a groove and data in the range 20–3.6 Å resolution. The molecular model is also shown. Figure obtained using *Chimera* (Pettersen *et al.*, 2004 ▸) with electron density cut at 1σ and zoned around atoms to 2 Å.

The first set of simulations used a cylindrical starting envelope of radius 48 Å, with the shrink-wrap algorithm applied every five iterations. All ten runs converged to the correct solution. The results are summarized in Table 3 ▸. The error metrics versus iteration for one of the runs are shown in Fig. 5 ▸(*a*), with final error metrics 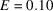 , 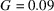 and 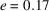 . The true and an example reconstructed electron density in one *c* repeat are shown in Fig. 6 ▸, and a good reconstruction is evident. The reconstructed electron density in the α-helical region of residues LEU 296 to PHE 317, together with the molecular structure, are shown in Fig. 7 ▸(*a*). The quality of the resulting map is sufficient for model building.

**Figure 8:**
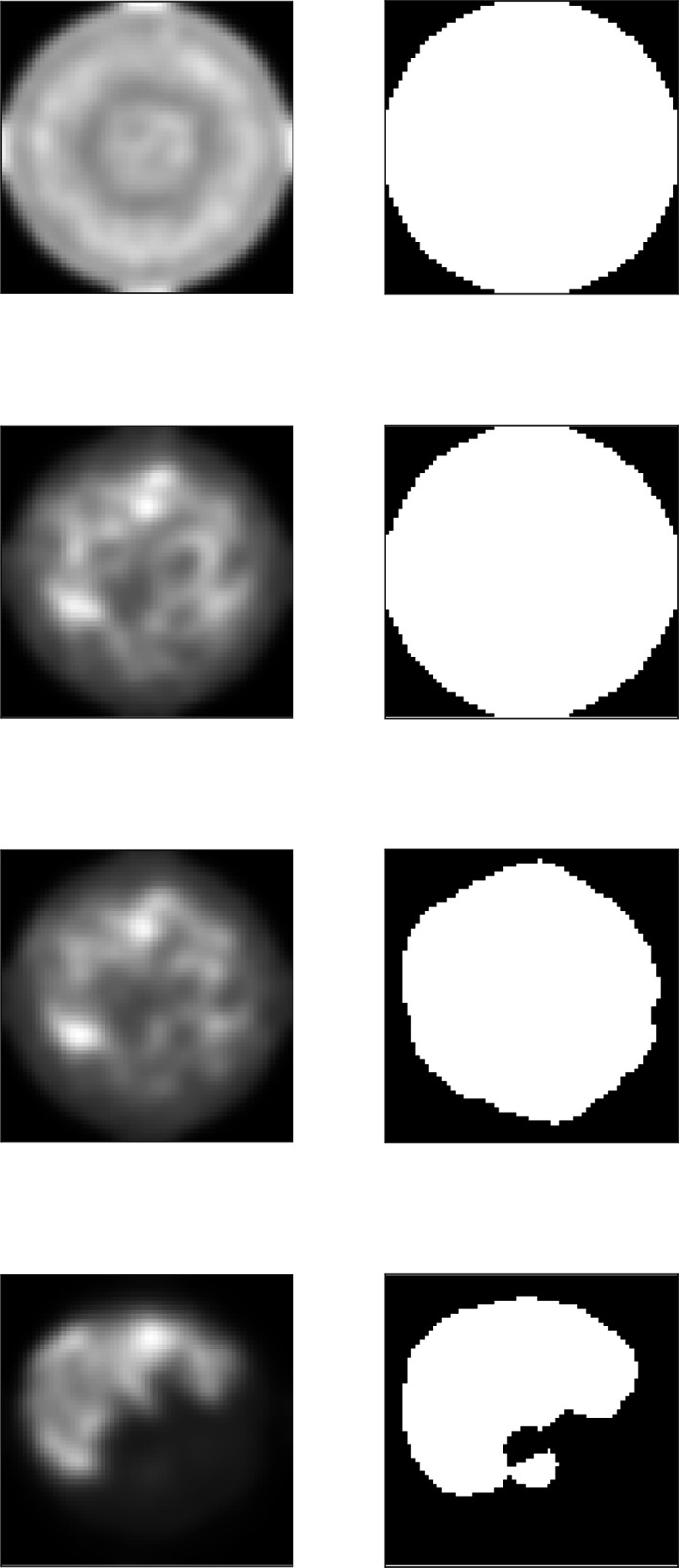
The projected untwisted electron density 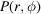 (left) and the corresponding 2D envelope 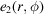 (right) at iterations 10, 100, 480 and 580 (top to bottom) for the RAD51 reconstruction starting with a cylindrical envelope.

As for the case of DNA, the shrink-wrap algorithm is surprisingly effective in evolving a cylindrical starting envelope into a helical (non-cylindrical) envelope to obtain a solution. The projected untwisted electron density 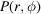 and the corresponding 2D envelope 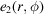 at different stages of the algorithm for a converged run are shown in Fig. 8 ▸. Starting with a cylindrical envelope, the density is initially approximately circularly symmetric and, subsequently, a non-symmetric density develops, resulting in a non-symmetric projected envelope. With the resulting non-cylindrical envelope, the problem is well determined and the envelope and the density converge to the correct solution.

Since the low-resolution diffraction data are important in defining the envelope, and are not necessarily easily measured, we investigated the effect of removing more of the low-resolution data. Reconstructions were attempted using the same protocol as above, but with intensity data between 20 and 3.6 Å resolution. In this case, good reconstructions were not obtained (Table 3 ▸), and the envelope did not evolve away from a cylinder. This shows the importance of the low-resolution data for *ab initio* phasing with minimal envelope information.

In cases where the algorithm is not able to converge from a starting cylindrical envelope, starting with a small deviation from a cylindrical envelope may be sufficient to initiate successful action of the shrink-wrap algorithm. The presence of helical symmetry offers the opportunity to start with a slightly non-cylindrical envelope. This is the case even if the presence and size of any helical groove are unknown *a priori*. The idea is that starting with a small groove of the correct pitch is likely not to be inconsistent with the true molecular envelope, but may be sufficient to effectively start the shrink-wrap procedure.

**Figure 9:**
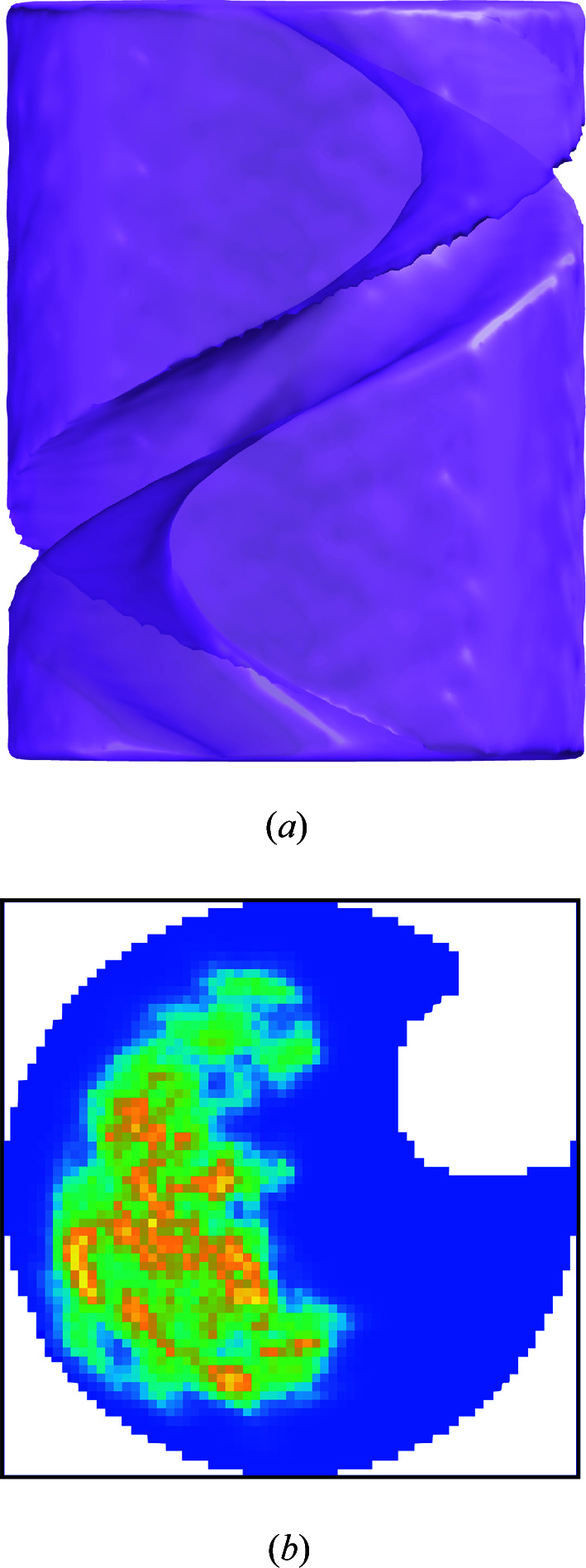
(*a*) Cylindrical envelope with a groove of 10% of the cylinder volume removed, used as a starting envelope for the RAD51 reconstruction, as described in the text. (*b*) The final reconstructed projected untwisted electron density for RAD51, and the initial 2D envelope in dark blue.

**Figure 10:**
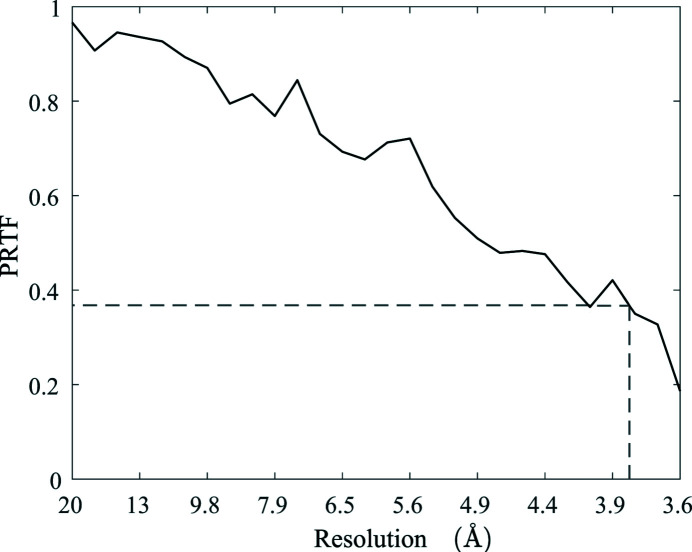
Phase retrieval transfer function (PRTF) as a function of inverse resolution. The resolution of the reconstruction is estimated where 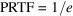 .

This idea was investigated by starting with an envelope consisting of a cylinder with a small helical groove of pitch 130 Å, containing 10% of the volume of the cylinder, removed, as shown in Fig. 9 ▸, followed by application of the shrink-wrap algorithm. The reconstruction algorithm was run using data in the range 20 to 3.6 Å. Good reconstructions were obtained (Table 3 ▸), and the error metrics versus iteration for an example run are shown in Fig. 5 ▸(*b*), the final error metrics being 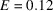 , 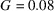 and 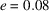 . Comparing Fig. 5 ▸(*b*) and Fig. 5 ▸(*a*) shows that the real-space error *e* reduces during the search phase in the former, but not in the latter. This may be due to the electron density evolving more rapidly towards the true density when it is constrained by the initial helical groove. An example of the reconstructed electron density in the region of residues LEU 296 to PHE 317 is shown in Fig. 7 ▸(*b*), and is of sufficient quality for model building. The resolution of the reconstruction is estimated by calculating the phase retrieval transfer function (PRTF) (Chapman *et al.*, 2006 ▸) 

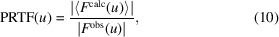

where *u* is inverse resolution, 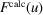 is the complex Fourier amplitude of the reconstruction, 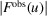 is the amplitude data, and the average is over the three best reconstructions in resolution shells. The PRTF is shown in Fig. 10 ▸. The resolution is estimated as where the PRTF falls to a value of 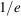 , which gives a resolution of the reconstructed density of about 3.8 Å, slightly less than the data resolution of 3.6 Å.

For RAD51, the helix is right-handed (*i.e.* 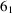 as determined from the crystal structure), but the hand may not be determinable from 1D crystal diffraction data. Therefore, the algorithm was also run with a left-handed helical envelope. The algorithm converged to a low Fourier-space error ( 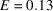 ), and inspection of the reconstructed density showed that it is the inversion of the true density. This is diagnostic of the incorrect hand, as described above.

## 6. Practical considerations

The results above show the basis and feasibility of *ab initio* phasing of diffraction data from 1D crystals. However, to put this idea into practice will involve overcoming a number of practical hurdles. Here we briefly consider some of these.

In nanocrystallography and single-particle imaging using XFELs, the first step after extracting specimen-diffraction-containing patterns (hit-finding), is orienting, or indexing, the patterns, *i.e.* determining the section through reciprocal space that a particular pattern represents (Shneerson *et al.*, 2008 ▸). This is necessary because the specimen orientation is usually not known *a priori* and needs to be determined from the recorded diffraction. In nanocrystallography with 3D crystals, this is referred to as indexing, as orienting a pattern in reciprocal space is equivalent to assigning Miller indices to the spots on the pattern. The presence of sharp Bragg reflections, even if they are few in number, substantially eases this problem, and successful indexing is now fairly routine in most cases (White *et al.*, 2012 ▸). For single-particle imaging (SPI), where the signal level above the background is low, the problem is more challenging. In particular, the continuous nature of the diffraction in SPI means that the problem is not fundamentally a discrete one, as it is for the crystalline case. However, approaches such as the expand–maximize–compress (EMC) method, in which a classification of the patterns is iteratively updated based on a probabilistic measure of the overall fit of the full data set, are able to orient large collections of weak patterns (Ayyer *et al.*, 2016 ▸).

Similarly to the above cases, the diffraction patterns from a 1D crystal will need to be oriented in reciprocal space prior to merging to increase the SNR to useable levels. We briefly consider this orientation problem in the likely experimental geometry for diffraction from 1D crystals.

**Figure 11:**
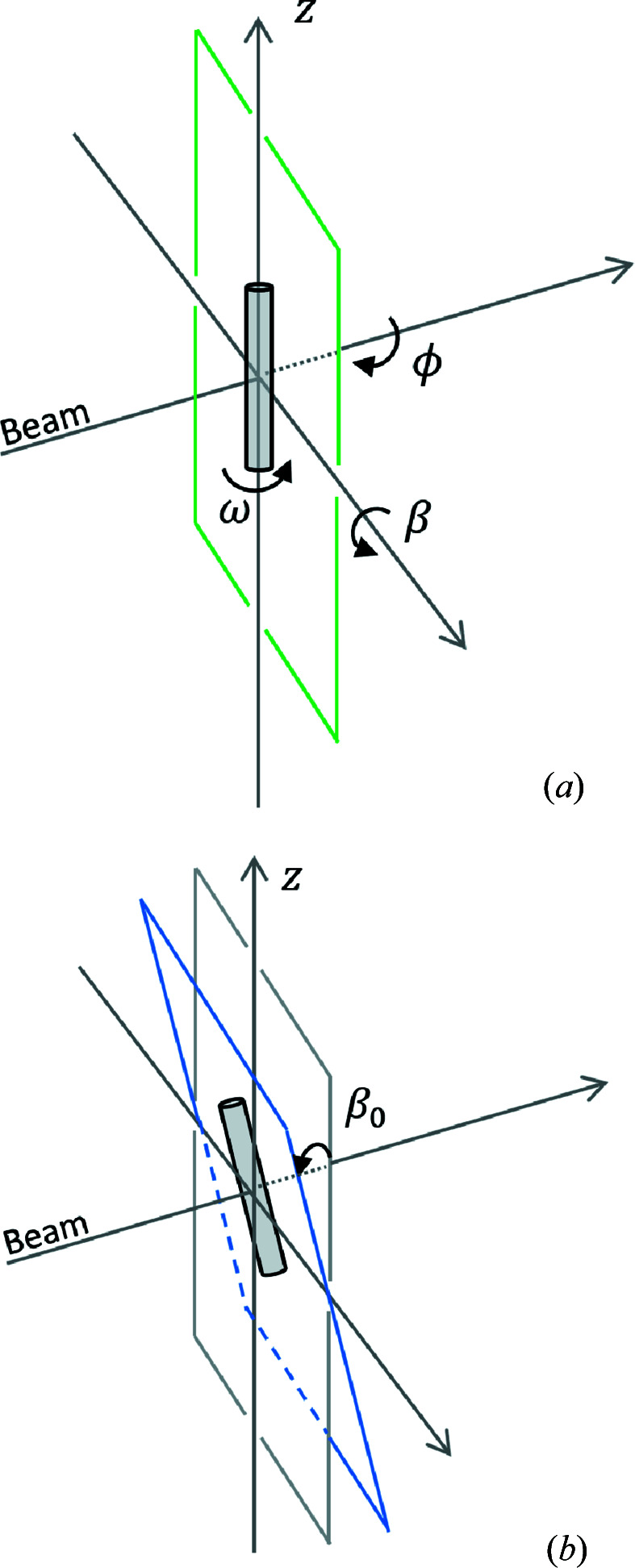
(*a*) Orientation angles of the 1D crystal in relation to the beam. (*b*) A 1D crystal on a fixed-target support with tilt β_0_.

The orientation of a diffraction pattern in reciprocal space corresponds directly to the orientation of the 1D crystal in real space (the laboratory coordinate frame). Following the conventional description in fiber diffraction, we define the orientation of the 1D crystal by the angles 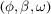 (Millane, 2010 ▸; Wojtas *et al.*, 2017 ▸), where ϕ is the rotation of the crystal axis (which we denote the *z* axis) about the incident X-ray beam, β is the tilt of the crystal axis out of the plane normal to the X-ray beam, and ω is the rotation of the crystal about its axis, as shown in Fig. 11 ▸(*a*). The angles 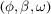 determine the 2D spherical section of reciprocal space that is sampled by the 2D diffraction pattern recorded from a single 1D crystal. Note that for a fiber specimen ω is randomized, but for the case considered here, each 1D crystal will give data for a single value of ω.

**Figure 12:**
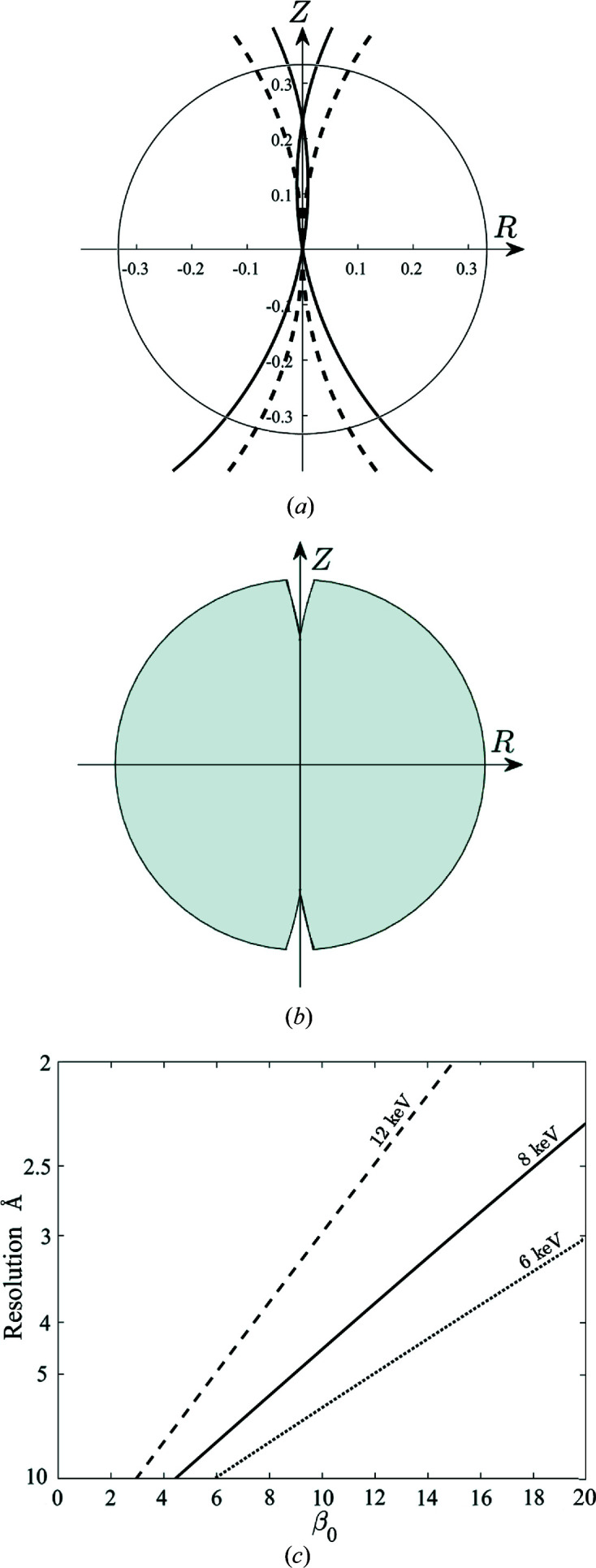
(*a*), (*b*) Coverage of reciprocal space in 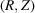 , for all values of Ψ. The circle indicates 3 Å resolution. (*a*) Coverage is to the left and right of the dashed (for β = 0°) and solid (for β = 10°) lines for a photon energy of 8 keV (λ = 1.55 Å). For β = 10° the lens-shaped region about the *Z* axis is also excluded. (*b*) Coverage for all tilt angles 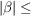 10°. (*c*) Maximum resolution for 100% completeness as a function of 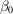 for photon energies 12 keV (λ = 1.03 Å) (dashed line), 8 keV (λ = 1.55 Å) (solid line), 6 keV (λ = 2.07 Å) (dotted line).

Consider first the case of fixed-target delivery of the specimen in the sample chamber, such as, for example, graphene supports mounted on a silicon chip, as this method has the potential for very low background diffraction compared with liquid-jet delivery (Roedig *et al.*, 2017 ▸; Seuring *et al.*, 2018 ▸). We denote by 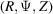 cylindrical polar coordinates in reciprocal space, with *Z* conjugate to *z* in real space. The 1D crystals are assumed to lie flat on the support, and to adopt a variety of rotations ω about their axis (which is in the plane of the support), with enough values of ω to sufficiently sample the transform in Ψ. For a particular value of tilt β, the diffraction patterns over the range of ω values fill a region in reciprocal space that is a cylinder (within the overall resolution limits), *i.e.* all values of Ψ, except for a region around the *Z* axis that depends on the tilt. For zero tilt, two cone-like regions are excluded, and for non-zero tilt two cone-like regions and an additional lens-shaped region at low resolution about the *Z* axis are excluded, as shown in Fig. 12 ▸(*a*).

Consider now a fixed-target support that is tilted at angle 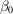 to the plane normal to the incident X-ray beam, as shown in Fig. 11 ▸(*b*). Since the molecules will adopt all values of ϕ on the support, all values of tilt β will be present on the interval 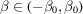 . Referring to Fig. 12 ▸(*b*), the corresponding diffraction frames will fill reciprocal space out to a resolution 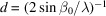 , with a small cone-like region around the *Z* axis missing at higher resolutions. For example, at a photon energy of 8 keV (λ = 1.55 Å) and 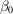 = 10°, reciprocal space is filled out to 4.5 Å resolution, and only a small region is missing at higher resolution, as shown in Fig. 12 ▸(*b*). In this case, for example, the shell between 4.5 and 3 Å resolution is approximately 90% complete. In summary, diffraction patterns recorded from 1D crystals that lie with random rotations on a planar support that is tilted relative to the incident X-ray beam will provide sufficient information to fill reciprocal space out to a specific resolution that depends on the tilt and the wavelength. The maximum resolution as a function of tilt and photon energy, for 100% completeness, is shown in Fig. 12 ▸(*c*).

In this scenario, each diffraction pattern from a single 1D crystal needs to be oriented in reciprocal space, *i.e.* the angles 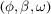 determined. This problem is intermediate in difficulty between the indexing problem for 3D crystals and the orientation problem for SPI. A proposed approach is as follows. Sections of the layer planes, *i.e.* layer lines, will be the strongest features, weakly present as curved lines in each pattern. Detection of these lines should allow the angle ϕ to be estimated, followed by, or in conjunction with, estimation of β. Note that determination of β is eased somewhat since it is known to lie in the interval 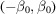 . Furthermore, since the tilt axis of the support is known, ϕ and β are highly correlated, up to a sign ambiguity in β. For example, if the tilt axis is defined as 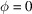 , then we have that 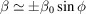 for an individual crystal. Since the patterns will be weak, it is likely that some sort of automated EMC-like algorithm will be needed to match features and determine ϕ and β. Once these two angles are determined, it is required to determine the angle ω for each pattern. This will be the most challenging step as there are no strong crystalline peaks. However, since there is only one parameter to be determined, it is less difficult than for the SPI case, and an EMC-like approach should be effective. Note that if the molecule has 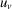 helical symmetry, then ω needs to be determined only on the interval 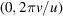 , rather than on the interval 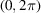 . Once the orientation angles are determined for each pattern, they can be mapped into reciprocal space and merged into a full 3D data set. Although angle determination and merging are described separately above, it is likely that they will be conducted together, *i.e.* consistency among merged patterns will be used to iteratively post-refine the orientation classification, as in the usual EMC approach.

A possible alternative specimen-delivery system is a microjet which can also give very low background diffraction and can orient high-aspect-ratio particles (Branden *et al.*, 2019 ▸). In this case, the pattern orientation approach would be similar to that described above, although it will be eased somewhat since both ϕ and β will be known to be within small ranges as determined by the orientation of the microjet. On the other hand, completeness of the data set will depend on the range of orientations that the particles adopt relative to the jet axis. If this range is narrow, then a few different jet tilts may be required to obtain a high degree of completeness.

A further consideration is the number of diffraction patterns likely to be required to generate a data set with sufficient SNR. The intensity of a Bragg reflection (or the mean intensity on a Bragg plane) is proportional to the square of the number of unit cells intersected by the beam, be it a 3D or a 1D crystal. Therefore, the data quality from a 1D crystal should be comparable with that from a 3D crystal with the same number of unit cells. Structure determination by serial femtosecond crystallography (SFX) has been successful with as few as about 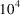 unit cells in the XFEL focus and an SNR of 1–3 in the highest-resolution shell, with as few as 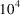 indexed patterns (Boutet *et al.*, 2012 ▸; Conrad *et al.*, 2015 ▸). The simulations above show that SNRs of the same order should be sufficient for *ab initio* phasing from 1D crystal data, so that a similar number of unit cells may be required. For a 1D crystal however, the number of unit cells in the beam is likely to be much smaller than in the 3D crystal case. Larger focal spots may be needed in order to intersect a sufficient number of unit cells. For example, with a *c* repeat of 50 Å and a 1 µm beam focus, there would be only about 200 unit cells in the beam. If the assembly length is smaller than the beam diameter then, obviously, the signal level is determined by this length rather than by the beam diameter. In any case, this implies that of the order of 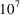 indexed diffraction patterns would be required. While this is a large number, there is reason to be cautiously optimistic. Improvements in low-background sample supports or other delivery systems will increase the achievable SNR of individual patterns and thence reduce the number of patterns required. Increases in XFEL pulse intensity and pulse rates, and improvements in sample scanning technology, will facilitate measuring and processing larger numbers of patterns. In summary, while collecting sufficient data from 1D crystals will be challenging, it is likely that various technological improvements over coming years will bring suitable data collection within reach.

A final consideration is the assumption that the assembly forms a perfect 1D crystal. In particular, as a result of the large aspect ratio and some degree of flexibility, the molecular axis may exhibit deviations from a straight line. Such distortions will affect the diffracted intensity at high resolution. The maximum resolution to which the data represent an exact 1D sampling of the transform of one repeat unit will be approximately equal to the mean deviation of the assembly axis from the mean axis of the crystal. The effect is analogous to that of mosaicity in 3D crystals which, however, does not fundamentally affect the resolution of the diffraction data if present to a small degree. The same will be the case for a 1D crystal, and small deviations from a straight axis can be tolerated or corrected for. However, the crystal would need to be reasonably well approximated by a rigid rod over the length of the crystal in the focal region. The resolution achievable in a particular case will be limited by the assembly flexibility, and the approach will be more suitable for stiff assemblies such as microtubules than for more flexible polymers, without the use of preparations that promote additional alignment of the axis.

## 7. Conclusions

Structure determination from 1D crystal diffraction offers some advantages for studies of fibrillar systems, in terms of circumventing the effects of cylindrical averaging and disorientation in traditional fiber diffraction analysis, and opportunities for *ab initio* phasing. Measuring diffraction from a single 1D crystal has been out of reach with conventional X-ray sources, but becomes feasible with XFEL sources. The phase problem for 1D crystals is better determined than for 3D crystals, and approaches that for SPI while offering greater signal levels. Previous theoretical results indicate that direct reconstruction should be possible if a slightly non-cylindrical envelope constraint can be applied.

The simulation results presented here indicate that, with realistic noise levels and missing data, starting with an unstructured cylindrical envelope and incorporation of a 2D helical shrink-wrap algorithm can allow direct reconstruction in favorable cases. Alternatively, using the same approach but starting with a small helical groove may also allow direct reconstruction. Implementation of a less constrained 3D shrink-wrap algorithm may further increase the radius of convergence. Although the results presented are promising, there are some significant practical hurdles that would need to be overcome for practical implementation of the approach. These include sample delivery, orienting the weak diffraction patterns, and measuring enough patterns to give a data set with sufficient SNR. However, current progress in measuring XFEL data from such systems, and likely upcoming improvements in sample delivery and XFEL technology, bode well for the potential of such an approach.

## Figures and Tables

**Table 1:** Reconstruction results for a simple object, for the three cases described in the text

Case	Cylindrical envelope	Positivity	Λ	Runs converged	Correct solutions	Average No. of iterations
1	Y	N	1	100/100	0/100	147
2	Y	Y	1	5/100	5/5	5302
3	N	N	1.05	30/100	30/30	456

**Table 2:** Reconstruction results for B-DNA

Relative groove volume	Λ	Shrink-wrap	Runs converged	Correct solutions	Average No. of iterations
0	1	N	0/10	–	–
0.05	1.05	N	0/10	–	–
0.10	1.11	N	10/10	10/10	700
0.20	1.25	N	10/10	10/10	300
0	–	Y	8/10	8/8	900

**Table 3:** Reconstruction results for RAD51

Cylindrical starting envelope	Data resolution (Å)	Runs converged	Correct solutions	Average No. of iterations	Average *E*	Average *G*	Average *e*
Y	40–3.6	10/10	10/10	700	0.14	0.10	0.12
Y	20–3.6	0/10	–	–	–	–	–
N	20–3.6	10/10	10/10	600	0.13	0.09	0.08
